# Alteration of starch hydrolyzing enzyme inhibitory properties, antioxidant activities, and phenolic profile of clove buds (*Syzygium aromaticum* L.) by cooking duration

**DOI:** 10.1002/fsn3.284

**Published:** 2015-09-23

**Authors:** Stephen A. Adefegha, Ganiyu Oboh, Sunday I. Oyeleye, Kolawole Osunmo

**Affiliations:** ^1^ Functional food and Nutraceutical UnitDepartment of BiochemistryFederal University of Technology P.M.B. 704Akure340001Nigeria

**Keywords:** Antioxidant, clove bud, cooking duration, diabetes mellitus, polyphenol, vitamin C

## Abstract

This study assessed the effect of cooking duration on starch hydrolyzing enzyme (*α*‐amylase and *α*‐glucosidase) activities, antioxidant (1,1‐diphenyl‐2 picrylhydrazyl [DPPH*], hydroxyl [OH*] radicals scavenging abilities and reducing power) properties, and phenolic profile of clove buds. Clove buds (raw) were cooked for 10 (SC_10_) and 20 min (SC_20_) and subsequently, their effects were assessed on enzyme activities, antioxidant properties, and phenolic profile. Inhibition of *α*‐amylase and *α*‐glucosidase activities and radicals scavenging abilities were altered by cooking in the trend; raw < SC_10_ > SC_20_, with IC_50_ values ranging from 0.25 to 0.52 mg/mL and 0.10 to 1.50 mg/mL respectively. HPLC phenolic profile of the clove buds revealed significant (*P* < 0.05) changes in the amount of chlorogenic acid, quercitrin, quercetin, and kaempferol at different cooking duration. Thus, cooking duration may alter the phenolic compositions and nutraceutical potentials of clove bud by activation and/or deactivation of redox‐active metabolites.

## Introduction

Phenolic compounds are plant secondary metabolites, which are synthesized by plant adaptation to several biotic and abiotic stress conditions such as cold, water, infections drought, temperature, and nutrient deficiencies (Dicko et al. 1995). They are widely present in vegetables, fruits, legumes, cereals, nuts, and beverages, which upon consumption have been reported to reduce the development of several human degenerative diseases such as coronary heart disease, cancer, hypertension, diabetes, osteoporosis, and neurodegenerative diseases (Pandey and Rizvi 2009; Adefegha et al. 2014). There are thousands of phenolic phytochemicals which can be divided into at least 10 different groups depending on their basic chemical structure. Flavonoids constitute a very important group because of their capacity to protect living organism against free radicals and oxygenated reactive species (ORS) produced during the metabolism of oxygen (Wach et al. 2007; Adefegha and Oboh 2013; Lima et al. 2014; Oboh et al. 2015).

Diabetes mellitus (DM) is a metabolic disorder that occurs as a result of inability of the body to secrete insulin, or to make use of the insulin produced, or both. Insulin deficiency can lead to chronic hyperglycemia with disturbances of carbohydrate, fat, and protein metabolism (Adefegha and Oboh 2012; Oboh et al. 2015). The two major forms of diabetes are insulin‐dependent diabetes mellitus (type‐1 diabetes) and noninsulin‐dependent diabetes mellitus (type‐2 diabetes). Both insulin‐dependent diabetes mellitus (IDDM) and noninsulin‐dependent diabetes mellitus (NIDDM) share one central feature which is elevation of blood sugar (glucose) levels. NIDDM is the most common form of diabetes, accounting for 90% of the cases. Based on the World Health Organization (WHO) report, the occurrence of NIDDM affects more than 170 million people worldwide and it is estimated that by the year 2030, the total number of people having diabetes will reach 366 million (Fonseca 2006).

Clove bud (*Syzygium aromaticum* L. Merr. & Perry) is an unopened flower bud growing on the tree belonging to the family of Myrtaceae. It is a commonly consumed spice that is used in the preparation of several delicacies in many homes, therefore makes it a precious and valuable spice of the world (Milind and Deepa 2011). Clove bud is deep brown in color and possess intense odor and slightly astringent. They are consumed as whole spices or ground into powder and mixed with diets containing cereals, legumes, nuts, fruits, vegetables, milk, and milk products (Adefegha and Oboh 2013). Aside from its use in culinary purposes, it is also used in folklore for the management and treatment of inflammation, liver damage, cancer, diabetes, and other oxidative stress–induced diseases for several centuries, probably due to the abundance of bioactive compounds such as volatile (e.g., eugenol) and nonvolatile (e.g., polyphenols, tannin, terpenes and triterpenes, and sterols) constituents (Atawodi et al. 2011; Milind and Deepa 2011).

Earlier studies in our laboratory have shown in part the rationale behind the use of clove bud in the management of some pathologies including diabetes and hypertension (Adefegha and Oboh 2012, 2013; Adefegha et al. 2014). However, cooking methods and duration were not put into consideration in these studies. In Nigeria, clove bud is consumed both in its raw and cooked forms. The duration of cooking may affect both the bioactive constituents and the biological activities of the plant foods. Thus, this work was designed to evaluate the effect cooking duration on the phenolic constituents, antioxidant properties, and interaction of clove bud extracts (raw and cooked) on key enzymes linked to type‐2 diabetes (*α*‐amylase and *α*‐glucosidase) in order to ascertain the best form by which clove bud can be used as nutraceutical and functional food.

## Material and Methods

### Sample collection and identification

The dried spice of clove bud was purchased at Oja Oba market in Akure metropolis, Nigeria. Authentication of the samples was carried out in the Department of Crop Soil and Pest Management, Federal University of Technology, Akure, Nigeria.

### Chemicals and reagents

Chemicals and reagents used such as thiobarbituric acid (TBA), 1,10‐phenanthroline, deoxyribose, gallic acid, Folin–Ciocalteau reagent were procured from Sigma‐Aldrich, Inc., (St. Louis, MO), trichloroacetic acid (TCA) was sourced from Sigma‐Aldrich, Chemie GmbH (Steinheim, Germany), hydrogen peroxide, methanol, sodium nitroprusside, Griess reagent, acetic acid, hydrochloric acid, sodium carbonate, aluminum chloride, potassium acetate, sodium dodecyl sulfate, iron (II) sulfate, potassium ferricyanide, and ferric chloride were sourced from BDH Chemicals Ltd., (Poole, UK). Except stated otherwise, all other chemicals and reagents were of analytical grades and the water was glass distilled.

### Sample preparation

Ten grams of clove buds powder was steam cooked in 200 mL of distilled water for 10 (SC_10_) and 20 min (SC_20_), respectively, while another 10 g was soaked raw (SR) in 200 mL of distilled water for 1 h and filtered alongside the cooked portion. The filtrate from SR, SC_10_, and SC_20_ was kept in the refrigerator for subsequent analysis.

### *α*‐Amylase inhibition assay

Raw and cooked clove bud extracts (0–200 *μ*L) and 500 *μ*L of 0.02 mol/L sodium phosphate buffer (pH 6.9 with 6 mmol/L NaCl) containing porcine pancreatic *α*‐amylase (EC 3.2.1.1) (0.5 mg/mL) were incubated at 25°C for 10 min. To each tube, 500 *μ*L of 1% soluble potato starch solution in 0.02 mol/L sodium phosphate buffer (pH 6.9 with 6 mmol/L NaCl) was then added to each tube. The reaction mixture was incubated at 25°C for 10 min and stopped with 1.0 mL of dinitrosalicylic acid color reagent. Thereafter, the mixture was incubated in a boiling water bath for 5 min, and cooled to room temperature. The reaction mixture was then diluted with 10 mL of distilled water, and absorbance was measured at 540 nm. The *α*‐amylase inhibitory activity was calculated and expressed as percentage inhibition (Worthington 1993) using the formula below:

$$\left(\%\right)\;\text{Inhibition}=\left(\frac{{\text{Abs}}_{\mathrm{ref}}-{\text{Abs}}_{\mathrm{sam}}}{{\text{Abs}}_{\mathrm{ref}}}\right)\times 100$$

where Abs_ref_ is the absorbance without sample and Abs_sam_ is the absorbance of the extract.

### *α*‐Glucosidase inhibition assay

Raw and cooked clove bud extracts (0–200 *μ*L) and 100 *μ*L of *α*‐glucosidase (EC 3.2.1.20) solution in 0.1 mol/L phosphate buffer (pH 6.9) were incubated at 25°C for 10 min. Fifty microliters of 5 mmol/L *p*‐nitrophenyl‐*α*‐d‐glucopyranoside solution in 0.1 mol/L phosphate buffer (pH 6.9) and 3 mmol/L glutathione was added. The mixtures were incubated at 25°C for 5 min, before reading the absorbance at 405 nm in the spectrophotometer. The *α*‐glucosidase inhibitory activity was expressed as percentage inhibition (Apostolidis et al. 2007). The formula for *α*‐glucosidase inhibition (%) is as follows:

$$\left(\%\right)\;\text{Inhibition}=\left(\frac{{\text{Abs}}_{\mathrm{ref}}-{\text{Abs}}_{\mathrm{sam}}}{{\text{Abs}}_{\mathrm{ref}}}\right)\times 100$$

where Abs_ref_ is the absorbance without sample and Abs_sam_ is the absorbance of the extract.

### Free radical scavenging ability

The free radical scavenging ability of the extracts against 1,1‐diphenyl‐2‐picrylhydrazyl **(**DPPH) free radical was evaluated as described by Gyamfi et al. (1999). Briefly, appropriate dilutions of the extracts (1 mL) were mixed with 1 mL of 0.4 mmol/L DPPH radicals in methanolic solution. The mixture was left in the dark for 30 min, and the absorbance was taken at 516 nm. The control was carried out by using 2 mL DPPH solution without the test samples. The DPPH free radical scavenging ability was subsequently calculated as percentage of the control using the following formula:

$${\text{DPPH}}^{*}\text{scavenging ability}\;\left(\%\right)=\left(\frac{{\text{Abs}}_{\mathrm{ref}}-{\text{Abs}}_{\mathrm{sam}}}{{\text{Abs}}_{\mathrm{ref}}}\right)\times 100$$

where Abs_ref_ is the absorbance without samples extract and Abs_sam_ is the absorbance of extracts.

### Fenton's reaction (OH radical scavenging ability)

The ability of the extracts to prevent Fe^2+^/H_2_O_2_‐induced decomposition of deoxyribose was carried out using the method of Halliwell and Gutteridge (1981). Briefly, varied dilutions of the extracts were added to a reaction mixture containing 120 *μ*L of 20 mmol/L deoxyribose, 400 *μ*L of 0.1 mol/L phosphate buffer, 40 *μ*L of 20 mmol/L hydrogen peroxide, and 40 *μ*L of 500 *μ*mol/L FeSO_4_, and the volume was made up to 800 *μ*L with distilled water. The reaction mixture was incubated at 37°C for 30 min, and the reaction was then stopped by the addition of 0.5 mL of 2.8% TCA, this was followed by the addition of 0.4 mL of 0.6% TBA solution. The test tubes were subsequently incubated in boiling water for 20 min. The absorbance was measured at 532 nm in spectrophotometer. The percentage (%) hydroxyl radical scavenging ability was subsequently calculated as follows:

$${\text{OH}}^{*}\text{scavenging ability}\;\left(\%\right)=\left(\frac{{\text{Abs}}_{\mathrm{ref}}-{\text{Abs}}_{\mathrm{sam}}}{{\text{Abs}}_{\mathrm{ref}}}\right)\times 100$$

where Abs_ref_ is the absorbance without samples extract and Abs_sam_ is the absorbance of extract.

### Determination of reducing power

The reducing property of the extracts was determined by assessing the ability of the extracts to reduce FeCl_3_ solution as described by Oyaizu (1986). A 500‐*μ*L aliquot of the extract was mixed with 2.5 mL of 200 mmol/L sodium phosphate buffer (pH 6.6) and 2.5 mL of 1% potassium ferricyanide. The mixture was incubated at 50°C for 20 min and then 2.5 mL of 10% trichloroacetic acid was added. This mixture was centrifuged at 650 rpm for 10 min. Five milliliters of the supernatant was mixed with an equal volume of water and 1 mL of 0.1% ferric chloride. The absorbance was measured at 700 nm and ferric reducing power was subsequently calculated and expressed as ascorbic acid equivalent (AAE).

### Quantification of compounds by high‐performance liquid chromatography coupled with diode array detection (HPLC‐DAD)

Reverse phase chromatographic analyses were carried out under gradient conditions using C_18_ column (4.6 mm × 150 mm) packed with 5‐*μ*m diameter of the particles; the mobile phase was (A) acetonitrile:water (95:5, v/v) and (B) water:phosphoric acid (98:2, v/v), and the composition gradient was 5% of A until 10 min and changed to obtain 20%, 40%, 60%, 70%, and 100% of A at 20, 30, 40, 50, and 60 min, respectively, following the method described by Kamdem et al. (2013) with slight modifications. *Syzygium aromaticum* extracts were analyzed at a concentration of 15 mg/mL. The presence of 10 antioxidant compounds were investigated, namely, gallic acid, catechin, chlorogenic acid, caffeic acid, ellagic acid, quercetin, quercitrin, luteolin, kaempferol, and rutin. Identification of these compounds was performed by comparing their retention time and UV absorption spectrum with those of the commercial standards. The flow rate was 0.6 mL/min, injection volume 50 *μ*L, and the wavelengths were 271 nm for gallic acid, 280 nm for catechin, 327 nm for chlorogenic, ellagic, and caffeic acids, and 365 nm for rutin, luteolin, quercitrin, kaempferol, and quercetin. The samples and mobile phase were filtered through 0.45‐*μ*m membrane filter (Millipore) and then degassed by ultrasonic bath prior to use. Stock solutions of standards references were prepared in the HPLC mobile phase at a concentration range of 0.030–0.250 mg/mL for catechin, quercetin, quercitrin, luteolin, kaempferol, and rutin, and 0.020–0.300 mg/mL for gallic, chlorogenic, caffeic, and ellagic acids. The chromatography peaks were confirmed by comparing its retention time with those of reference standards and by DAD spectra (200–500 nm). Calibration curve was determined as follows: for gallic acid: *Y* = 13,568*x* + 1275.9 (*r* = 0.9996); catechin: *Y* = 11,985*x* + 1178.3 (*r* = 0.9998); epicatechin: *Y* = 12,643*x* + 1263.7 (*r* = 0.9995); chlorogenic acid: *Y* = 13,064*x* + 1187.6 (*r* = 0.9993); caffeic acid: *Y* = 12,876*x* + 1297.4 (*r* = 0.9997); ellagic acid: *Y* = 12,631*x* + 1243.9 (*r* = 0.9998); rutin: *Y* = 13,267*x* + 1265.8 (*r* = 0.9999); isoquercitrin: *Y* = 13,496*x* + 1273.2 (*r* = 0.9996); quercitrin: *Y* = 12,730*x* + 1308.5 (*r* = 0.9996); kaempferol: *Y* = 12,745*x* + 1289.5 (*r* = 0.9998); and quercetin: *Y* = 12,895*x* + 1362.7 (*r* = 0.9994). All chromatography operations were carried out at ambient temperature and in triplicate. The limit of detection (LOD) and limit of quantification (LOQ) were calculated based on the standard deviation of the responses and the slope using three independent analytical curves, as defined by Boligon et al. (2013). LOD and LOQ were calculated as 3.3 and 10 *σ*/S, respectively, where *σ* is the standard deviation of the response and S is the slope of the calibration curve.

### Determination of total phenol content

The total phenol content was determined according to the method of Singleton et al. (1999). Briefly, appropriate dilution of the raw and processed clove bud extracts were oxidized with 2.5 mL of 10% Folin–Ciocalteau reagent (v/v) and neutralized by 2.0 mL of 7.5% sodium carbonate. The reaction mixture was incubated for 40 min at 45°C and the absorbance was measured at 765 nm in the spectrophotometer. The total phenol content was subsequently calculated and presented as gallic acid equivalent (GAE).

### Determination of total flavonoid content

The total flavonoid content of the extracts was determined using a slightly modified method reported by Meda et al. (2005). Briefly, 0.5 mL of appropriately diluted extracts were mixed with 0.5 mL methanol, 50 *μ*L of 10% AlCl_3_, 50 *μ*L of 1 mol/L potassium acetate, and 1.4 mL water, and allowed to incubate at room temperature for 30 min. The absorbance of the reaction mixture was subsequently measured at 415 nm and the total flavonoid content was calculated as quercetin equivalent (QE).

### Determination of vitamin C content

Vitamin C content of the water extracts was determined using the method of Benderitter et al. (1998). Seventy‐five microliters of DNPH (2 g dinitrophenyl hydrazine, 230 mg thiourea, and 270 mg CuSO_4_·5H_2_O in 100 mL of 5 mol/L H_2_SO_4_) was added to 500 *μ*L of reaction mixture (300 *μ*L of extract with 100 *μ*L 13.3% TCA and water). The reaction mixtures were subsequently incubated for 3 h at 37°C, then 0.5 mL of 65% H_2_SO_4_ (v/v) was added to the medium, followed by absorbance measurement at 520 nm. The vitamin C content of the extracts was subsequently calculated as AAE.

### Data analysis

The results of the three replicates were pooled and expressed as mean ± standard deviation (SD). Student's *t*‐test, one‐way analysis of variance (ANOVA), and least significance difference (LSD) were carried out (Zar 1984). A *P *≤* *0.05 was considered statistically significant. EC_50_ was determined using linear regression analysis.

## Results

**Figure 1:**
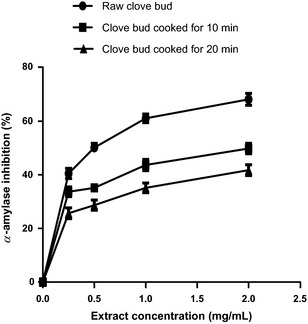
*α*‐Amylase inhibition of clove bud extracts. Values represent mean ± standard deviation of triplicate readings (*N* = 3).

**Figure 2:**
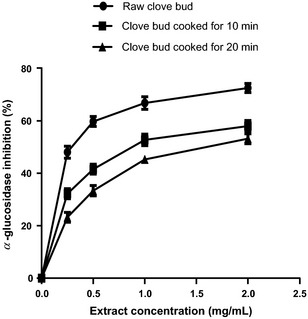
*α*‐Glucosidase inhibition of clove bud extracts. Values represent mean ± standard deviation of triplicate readings (*N* = 3).

The result of the *α*‐amylase inhibitory effect of the raw and processed clove bud extracts is presented in Figure 1. Clove bud (raw and cooked) extracts inhibited *α*‐amylase activity in a dose‐dependent manner and the EC_50_ values are reported in Table 1. The result revealed that the extract of clove bud cooked for 10 min (SC_10_) had the highest *α*‐amylase inhibitory activity (IC_50_ = 0.25 mg/mL). The effect of cooking duration on the *α*‐amylase inhibitory activity clove bud follows the trend; raw < SC_10_ > SC_20_ with IC_50_ values ranging from 0.25 to 0.50 mg/mL. Also, *α*‐glucosidase inhibitory activity (Fig. 2) of the extract from the clove bud cooked for 10 min had the highest *α*‐glucosidase inhibitory activity (IC_50_ = 0.25 mg/mL). The trend (raw < SC_10_ > SC_20_) observed on the effect of cooking duration on the *α*‐glucosidase inhibitory activity of clove buds is similar to that of the *α*‐amylase inhibitory effect of the clove buds.

**Table 1:** EC_50_ (mg/mL) values for the radicals (DPPH*, OH*) scavenging abilities, *α*‐amylase and *α*‐glucosidase inhibitory activities of raw and cooked (for 10 min and 20 min) clove buds

Parameter	Raw	Cooked for 10 min	Cooked for 20 min
DPPH* scavenging ability	1.40 ± 0.05^a^	1.10 ± 0.02^b^	1.50 ± 0.04^c^
OH* scavenging ability	0.15 ± 0.01^a^	0.10 ± 0.00^b^	0.13 ± 0.02^c^
*α*‐Amylase inhibition	0.48 ± 0.04^a^	0.25 ± 0.06^b^	0.50 ± 0.06^c^
*α*‐Glucosidase inhibition	0.50 ± 0.02^a^	0.25 ± 0.04^b^	0.52 ± 0.03^c^

Values represent mean ± standard deviation of triplicate readings (*n* = 3).

Values with the same superscript letter on the same row are not significantly different (*P *>* *0.05).

**Figure 3:**
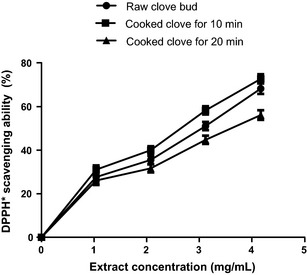
The effect of cooking time DPPH* scavenging ability of clove bud extracts. Values represent mean ± standard deviation of triplicate readings (*N* = 3).

**Figure 4:**
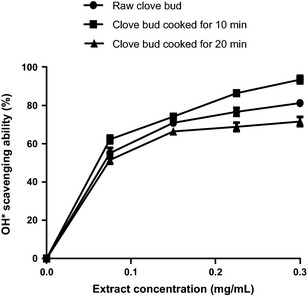
OH* scavenging ability of clove bud extracts. Values represent mean ± standard deviation of triplicate readings (*N* = 3).

The DPPH* scavenging ability of raw and cooked (10 and 20 min cooked) clove bud extracts and the extract concentration that scavenged 50% (EC_50_) are presented in Figure 3 and Table 1 respectively. The results revealed that SC_10_ (EC_50_ = 1.10 mg/mL) had the highest DPPH* scavenging ability, followed by raw (EC_50_ = 1.40 mg/mL) clove bud extract, while SC_20_ (EC _50_ = 1.50 mg/mL) had the least DPPH* scavenging ability. Furthermore, OH* scavenging ability of the clove bud extracts (Fig. 6) followed the similar trend in a concentration‐dependent manner (0–0.30 mg/mL) with SC_10_ (EC_50_ = 0.15 mg/mL) having the highest OH* scavenging ability (Table 2). The ferric reducing antioxidant power (FRAP) of the extracts were assessed based on their ability to reduce Fe^3+^ to Fe^2+^. The result of the reducing property of the clove buds extracts (raw, SC_10_, and SC_20_) is presented as AAE. SC_10_ had significantly (*P *<* *0.05) higher ferric reducing antioxidant properties than the raw and SC_20_ (Fig. 4).

**Table 2:** Phenolic composition of *Syzygium aromaticum* extracts

Compounds	*Syzygium aromaticum*			LOD (*μ*g/mL)	LOQ ( *μ*g/mL)
	Raw (mg/g)	10 min (mg/g)	20 min (mg/g)		
Gallic acid	14.07 ± 0.02^a^	8.03 ± 0.01^a^	8.19 ± 0.03^a^	0.015	0.053
Catechin	1.85 ± 0.03^b^	1.69 ± 0.01^b^	1.16 ± 0.01^b^	0.030	0.098
Chlorogenic acid	20.13 ± 0.01^c^	15.02 ± 0.02^c^	17.82 ± 0.02^c^	0.009	0.031
Caffeic acid	13.98 ± 0.03^a^	13.76 ± 0.03^d^	13.27 ± 0.01^d^	0.026	0.084
Ellagic acid	1.61 ± 0.02^b^	1.57 ± 0.01^b^	8.59 ± 0.01a	0.013	0.049
Rutin	9.11 ± 0.01^d^	13.92 ± 0.01^d^	3.41 ± 0.03^e^	0.007	0.023
Quercitrin	30.26 ± 0.03^e^	24.01 ± 0.03^e^	20.47 ± 0.02^f^	0.018	0.059
Quercetin	29.71 ± 0.02^e^	21.86 ± 0.01^f^	19.08 ± 0.01^f^	0.010	0.034
Kaempferol	20.47 ± 0.01^c^	21.73 ± 0.02^f^	20.95 ± 0.03^f^	0.036	0.119
Luteolin	9.28 ± 0.05^d^	14.05 ± 0.01^d^	8.57 ± 0.02^a^	0.014	0.045

Results are expressed as mean ± standard error of mean (SEM) of three determinations. Averages followed by different superscript letters differ by Tukey test at *P* < 0.05.

**Figure 5:**
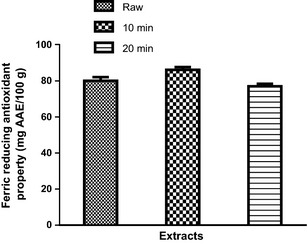
Ferric reducing antioxidant properties of clove bud extracts. Values represent mean ± standard deviation of triplicate readings (*N* = 3).

**Figure 6:**
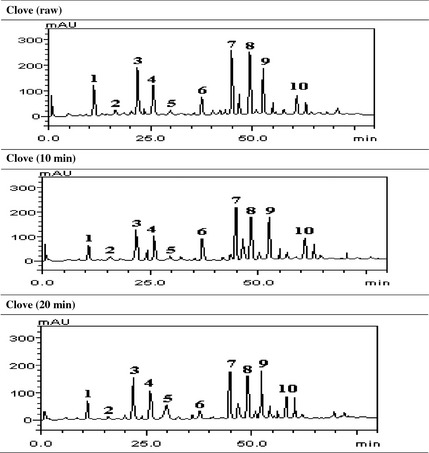
Representative high‐performance liquid chromatography profile of *Syzygium aromaticum* extracts. Gallic acid (peak 1), catechin (peak 2), chlorogenic acid (peak 3), caffeic acid (peak 4), ellagic acid (PEAK 5), rutin (peak 6), quercitrin (peak 7), quercetin (peak 8), kaempferol (peak 9), and luteolin (peak 10).

**Figure 7:**
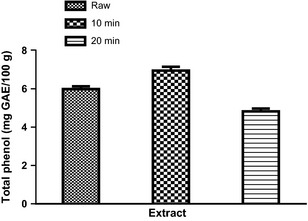
Total phenol content of the clove bud extracts. Values represent mean ± standard deviation of triplicate readings (*N* = 3).

**Figure 8:**
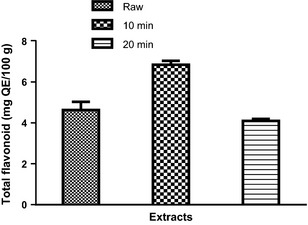
Total flavonoid content of the clove bud extracts. Values represent mean ± standard deviation of triplicate readings (*N* = 3).

HPLC phenolic fingerprinting of raw, SC_10_, and SC_20_ revealed the presence of gallic acid (*t*_R_ = 11.07 min; peak 1), catechin (*t*_R_ = 16.29 min, peak 2), chlorogenic acid (*t*_R_ = 21.83 min; peak 3), caffeic acid (*t*_R_ = 25.01 min; peak 4), ellagic acid (*t*_R_ = 29.76 min; peak 5), rutin (*t*_R_ = 37.54 min; peak 6), quercitrin (*t*_R_ = 44.98 min; peak 7), quercetin (*t*_R_ = 48.61 min; peak 8), kaempferol (*t*_R_ = 54.11 min; peak 9), and luteolin (*t*_R_ = 61.27 min; peak 10) (Fig. 5 and Table 2). The phenolic acids are gallic acid, chlorogenic acid, ellagic acid, caffeic acid, while the flavonoids present are (−) catechin, rutin, quercitrin, quercetin, kaempferol, and luteolin. Rutin, kaempferol, and luteolin values (Table 2) are in the trend: SC_10_ > SC_20_, while quercitrin, quercetin, and catechin are in the trend: raw > SC_10_ > SC_20_ (Table 2). Furthermore, the phenolic acids (gallic and chlorogenic acids) are in the trend: raw > SC_10_ < SC_20_. The result showed that there was no significant (*P *>* *0.05) difference in ellagic acid content of the raw and SC_10_ but higher in SC_20_. The result of the total phenol and flavonoid contents of the clove bud extracts (raw, SC_10_, and SC_20_) are presented in Figures 6 and 7 respectively. The results indicated that extract from SC_10_ had significantly (*P* < 0.05) higher total phenol and flavonoid contents than the raw clove bud extracts, while SC_20_ had the least. Conversely, the result of the vitamin C content revealed a significant (*P* < 0.05) decrease with increase in cooking duration. The trend (raw > SC_10_ > SC_10_) is observed as shown in Figure 8.

## Discussion

Cooking represents an indispensable prerequisite in obtaining safe and high‐quality food products. It confers better hygienic quality, palatability, and makes food more digestible (Adefegha and Oboh 2011). In this study, the effect of cooking duration was assessed on the antidiabetic potentials of clove buds via its inhibition on amylase and glucosidase activities, antioxidant (radicals [DPPH* and OH*] scavenging and FRAP) effects, phenolic profiling, as well as total phenolics (phenol and flavonoid) and vitamin C contents.

Inhibition of enzymes related to carbohydrate hydrolysis (*α*‐amylase and *α*‐glucosidase) has been accepted as one of the therapeutic approach in the management of type‐2 diabetes. The inhibition of *α*‐amylase and *α*‐glucosidase delay carbohydrate digestion thereby reduces rate of glucose production and consequently causes reduction in postprandial hyperglycemia especially in patients with type‐2 diabetes (Shim et al. 2003). From this study, all the extracts inhibited *α*‐amylase and *α*‐glucosidase activities in concentration‐dependent manner (0–2 mg/mL) and these biological effects (Figs. 1, 2) may be attributed to the presence of phenolic compounds such as phenolic acids and flavonoids (Table 2). Flavonoids such as kaempferol, rutin, and luteolin (which were found to be higher in SC_10_) have been reported to reduce the risk of type‐2 diabetes probably due to their stronger inhibition of *α*‐amylase and *α*‐glucosidase (Kim et al. 2000; Knekt et al. 2002; Oboh et al. 2015). It is worth noting that SC_20_ had the lowest *α*‐amylase and *α*‐glucosidase inhibitory effects and these may have resulted from the damage/loss of physiologically active phytochemicals through prolonged cooking duration as evidence in the phenolic contents (Figs. 7, 8) that have *α*‐amylase and *α*‐glucosidase inhibitory activities. Nevertheless, the determined *α*‐amylase inhibitory activity of the clove buds agreed with earlier reports of plant phytochemicals from some spices such as *Allium* spp., red and white ginger inhibitory effects on *α*‐amylase and *α*‐glucosidase activity in vitro (Nickavar and Yousefian 2009; Oboh et al. 2010).

Free radicals have been reported to play a major role in diabetic complications (Mohamed et al. 1999). Hence, steady consumption of dietary antioxidants to boost the endogenous antioxidant defense system is one of the accepted practical approach by which free radical–mediated oxidative stress in DM may be curtailed. The antioxidative properties of plant secondary metabolites occur by diminishing the production of free radicals or by neutralizing and/or scavenging radicals produced during normal body metabolism or reducing/chelating the transition metals present in foods. More so, termination of chain initiation step via scavenging of reactive species has been considered to be one of the antioxidant mechanisms of action (Melo et al. 2006; Dastmalchi et al. 2007). The result of the DPPH free radical scavenging ability of raw and cooked clove bud extracts revealed that SC_10_ caused significant (*P* < 0.05) increase in the DPPH* scavenging ability compared to the raw clove buds, while prolonged SC_20_ led to the decline in the DPPH* scavenging ability. Similar trend of result was also observed in the OH* scavenging ability of raw and cooked clove buds, where SC_10_ significantly (*P* < 0.05) increased OH* scavenging ability than that of the raw extract, while the SC_20_ had the least OH* scavenging ability. The differences in the radicals scavenging abilities of the raw and cooked clove buds could be as a result of destruction and/or creation of redox‐active phenolic compounds most especially flavonoid.

Reducing power is a novel antioxidation defense mechanism that involves electron and/or hydrogen atom transfers and reduces transition metals (Allhorn et al. 2005; Gocer and Gulcin 2011). This is because the ferric‐to‐ferrous ion reduction occurs rapidly in all reductants that have half‐reaction reduction potentials above that of Fe^3+^/Fe^2+^, the values of the FRAP assay could express the corresponding concentration of electron‐donating antioxidants. The trend of the result of the reducing power of the raw and cooked clove buds followed similar order as the total phenolic contents. Thus, this study agrees with previous studies where strong correlation were established between antioxidants properties of plant foods and their phenolic content (Adefegha and Oboh 2011, 2012; Carlonia et al. 2013). However, the antioxidant effects of the raw clove buds may be altered by cooking duration via the activation and/or deactivation of redox‐active metabolites/phenolics during the heat processes.

The presence of phenolic compound has been reported to contribute immensely to the enhancement of protective potentials of plant and plant‐based food against some degenerative diseases (Boyer and Liu 2004). Flavonoids have also been reported to possess antioxidative properties with the ability to lower cellular oxidative stress—a major culprit in the pathogenesis of various neurodegenerative diseases, such as Alzheimer's disease, Parkinson's disease, and amyotrophic lateral sclerosis (Adefegha et al. 2014)—via radicals scavenging and metals chelating effects, activation of antioxidant enzymes, and inhibition of oxidases (Altunkaya and Gökmen 2009; Muthiah et al. 2012).

The phenolic compound identified and quantified in the different clove bud extracts (raw, SC_10_, and SC_20_) may indicate that the spice under investigation is rich in phytochemicals with possible physiological importance. Furthermore, the extracts contained rutin, quercitrin, quercetin, and kaempferol as the major flavonoid composition, while chlorogenic acid was found to be present as the major phenolic acid. From the result, catechin, quercitrin, and quercetin contents decreased with increase in cooking duration. This trend agrees with earlier studies where thermal processing was reported to cause reduction in flavonoid content of foods based on the magnitude and extent of heat and duration (Viña and Chaves 2008; Zhang et al. 2010). The effects of cooking duration on the phenolic contents (total phenol and flavonoid) presented in Figs. 7, 8 revealed that SC_10_ increased the total phenol and flavonoid contents when compared to that of raw clove buds. This could be attributed to the release of stored total phenolics in cell wall bound pectins by thermal processing and breakage in the supramolecular structures or softened the plant matrix (Bunea et al. 2008; Ferracane et al. 2008). However, decreased contents of total phenol and flavonoid were observed in SC_20_ when compared to that of SC_10_. This observation agreed with the assertion that prolonged cooking duration could lead to thermal destruction or heavy loss of polyphenols (El‐Sohaimy 2013). From this study, the effect of the cooking duration on the phenolic contents of clove buds followed the trend: raw < SC_10_ > SC_20_ (Figs. 7, 8).

**Figure 9:**
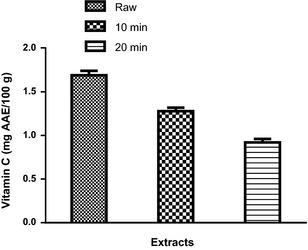
Vitamin C content of the clove bud extracts. Values represent mean ± standard deviation of triplicate readings (*N* = 3).

Plant foods including fruits, vegetables, and spices are good source of vitamin C (Babalola et al. 2010). Vitamin C is a prominent exogenous antioxidant that defends the body against free radicals, maintains blood vessel flexibility, improves blood circulation in the arteries, and protects against esophageal and stomach cancer (Block et al. 1992; Pandey and Rizvi 2009 Bashandy and Alwasel 2011). In this study, cooking significantly (*P* < 0.05) decreased the vitamin C content of clove buds. Hence, agreed with the report of Zhang and Hamauzu (2004) and Adefegha and Oboh (2011) that the loss in vitamin C contents with respect to cooking may be due to the thermal instability of vitamin C (ascorbic acid). Thus, the trend of the effect of cooking duration on the vitamin C content of clove buds observed in this study followed the order: raw > SC_10_ > SC_20_ as shown in Figure 9.

## Conclusion

The higher phenolic content, antioxidant properties, and inhibition of key enzymes (*α*‐amylase and *α*‐glucosidase) linked with type‐2 diabetes by SC_10_ compared to that of raw showed that consumption of moderate cooked clove bud with respect to time may indicate that potential antioxidant and antidiabetic agents are preserved and enhanced. However, prolonged cooking as in SC_20_ may lead to destruction of antioxidant and antidiabetic agents thereby reducing the nutritional quality and nutraceutical potentials of clove bud.

## Conflict of Interest

None declared.
